# Association between a body shape index and colorectal cancer in US population: a cross-sectional study based on NHANES

**DOI:** 10.3389/fnut.2025.1535655

**Published:** 2025-01-31

**Authors:** Hui Liu, Jialu Kang, Wei Liu, Yongqing Shen

**Affiliations:** ^1^Department of Internal Medicine Nursing, Faculty of Nursing, Hebei University of Chinese Medicine, Shijiazhuang, Hebei, China; ^2^Faculty of Nursing, Hebei University of Chinese Medicine, Shijiazhuang, Hebei, China

**Keywords:** ABSI, colorectal cancer, visceral fat, obesity, NHANES

## Abstract

**Background:**

Colorectal cancer (CRC) is linked to obesity, particularly visceral fat. A more accurate measure of visceral fat accumulation is offered by a body shape index (ABSI). Currently, the direct application of the ABSI to populations with varying ethnic backgrounds might be restricted. Moreover, there is less evidence about the correlation between ABSI and CRC among individuals from different ethnical backgrounds.

**Methods:**

A total of 40,998 individuals who took part in the National Health and Nutrition Examination Survey (NHANES) spanning from 2003 to 2023 were subjected to analysis. Logistic regression was utilized to examine the associations between the ABSI and the risk of CRC. In addition, restricted cubic spline curves (RCS) were utilized, and subgroup analyses along with interaction tests were also carried out. The receiver operating characteristic curve (ROC) was employed to predict the risk of CRC relying on various anthropometric indicators.

**Results:**

After adjusting for covariates, ABSI demonstrated a positive association with the incidence of CRC (OR = 1.03 [95% CI: 1.01–1.05], *p* = 0.018). Individuals in the upper quartile of ABSI exhibited a greater prevalence of CRC than those in the lower quartile (OR = 1.88 [95% CI: 1.19–2.96], *p* = 0.006). RCS analysis indicated a nonlinear correlation between ABSI and CRC (*P* for nonlinear = 0.030). Subgroup analysis indicated a notable interaction between age and BMI subgroups (interaction *p* < 0.05), and ROC curves indicated that the ABSI was effective in predicting CRC risk (AUC = 0.658), demonstrating good sensitivity, particularly in individuals under 60 years of age.

**Conclusion:**

A positive correlation exists between ABSI levels and the increased incidence of CRC among U.S. adults. This is especially true for people under 60 years of age (40–60 years), with a BMI below 25 kg/m^2^, and those with a BMI of 30 kg/m^2^ or beyond. ABSI can be used as a simple anthropometric predictor of CRC.

## Introduction

Colorectal cancer (CRC), a malignant neoplasm, is witnessing an escalating prevalence on a global scale. Its morbidity and mortality figures stand among the highest in different kinds of cancers. In 2022, there were more than 1.9 million newly reported cases, and the number of fatalities reached 904,000 (1). The prevalence of colorectal cancer is rising in various nations, including China and the United States, with an increasingly younger demographic being impacted (2, 3). In light of this context, it is crucial to aggressively investigate more effective methods for the prevention and screening of CRC to significantly diminish its incidence rate.

The incidence of obesity has witnessed exponential growth on a global scale as a result of lifestyle alterations, impacting over 650 million individuals (4). In particular, it has been established that visceral obesity is a significant risk factor for CRC development (5, 6). However, it has been shown that traditional measures of obesity, like the body mass index (BMI), possess limitations when it comes to evaluating the risk of CRC, as they are unable to accurately reflect the characteristics of fat distribution (7, 8). Furthermore, the precision of BMI in gauging obesity has been cast in doubt on account of its inbuilt limitations (9). A body shape index (ABSI) was developed by Krakauer and his fellow researchers in 2012. With waist circumference (WC) being its basis, it is adjusted corresponding to height and weight. Under certain height and weight circumstances, if the ABSI is raised, it is more prone to suggest a larger portion of visceral (abdominal) fat rather than peripheral tissue (10). The index has demonstrated superior efficacy in predicting the health consequences of obesity, as elevations in the ABSI correlate with substantial increases in mortality, underscoring the insufficiency of standard obesity indicators in assessing health hazards (11). The ABSI effectively addresses some constraints inherent in relying solely on overall or central obesity markers, hence facilitating the investigation of relationships with cancer risk (12). Nonetheless, to date, only a restricted number of research have investigated the association between ABSI and the risk of CRC.

Therefore, this study sought to elucidate the relationship between ABSI and the incidence of CRC utilizing individual data from the National Health and Nutrition Examination Survey (NHANES) database. The objective was to investigate this correlation and formulate efficient preventative and screening measures for the incidence of CRC.

## Materials and methods

### Data sources

**Figure 1:**
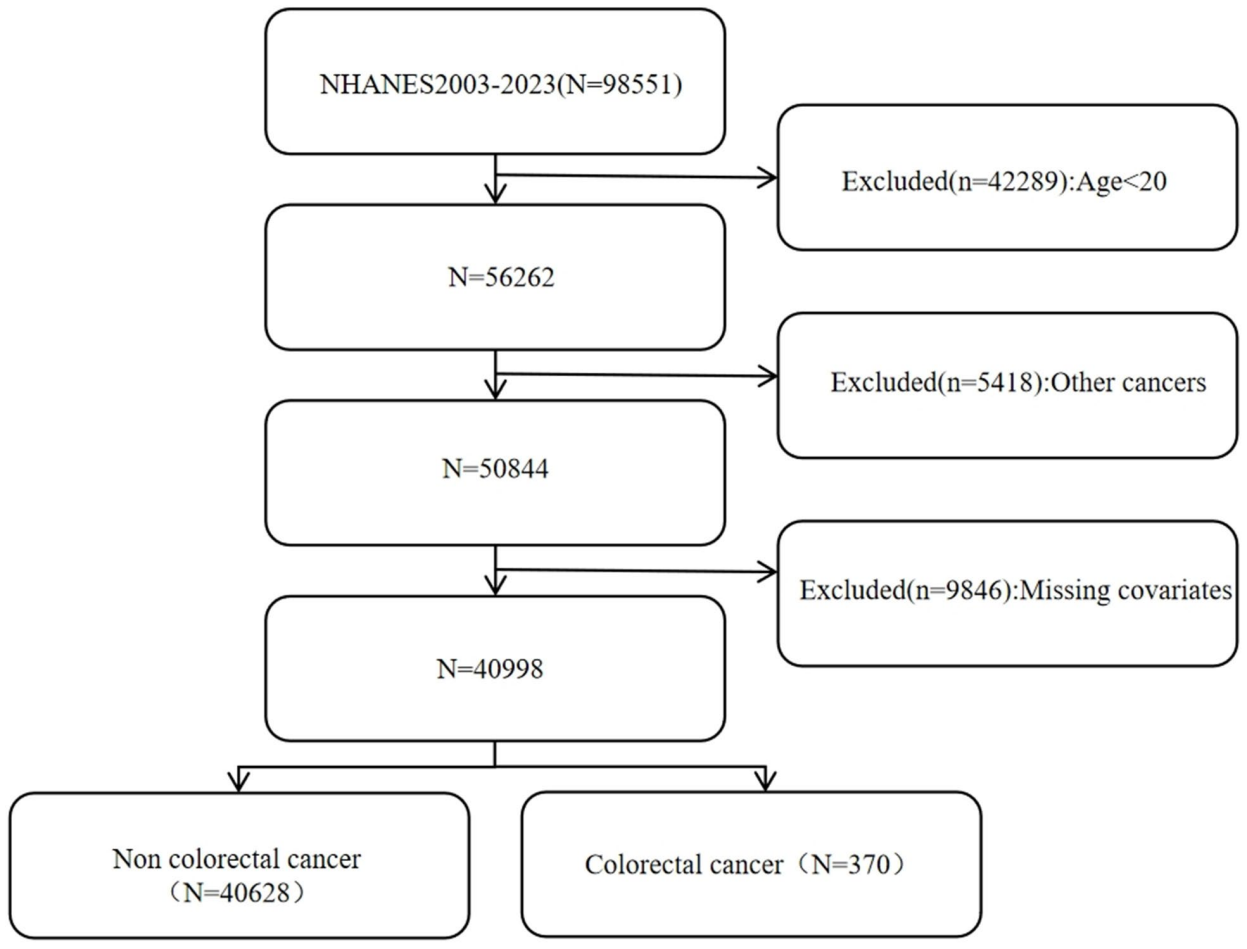
The flow chart of this study.

The NHANES is an ongoing cross-sectional survey that represents the nation as a whole. It employs a stratified, multistage random sampling approach and is conducted by the National Center for Health Statistics within the United States. Additionally, every participant has furnished written informed consent. All of the data can be conveniently retrieved from the NHANES website. This study analyzed 98,551 people from 2003 to 2023, removing individuals under 20 years of age, those with additional malignancies, and those missing critical covariate data. In total, 40,998 participants were incorporated into the study analysis according to stringent inclusion and exclusion criteria. The detailed steps of the specific inclusion process can be found in Figure 1.

### Definition of ABSI and colorectal cancer

In this study, the exposure variable was the ABSI. The researchers collected basic anthropometric measurements from the participants, such as WC, height, and BMI. The formula for calculating the ABSI is ABSI = WC/(BMI^⅔^ × height^½^) (10, 13), with WC and height in meters.

Colorectal cancer was identified based on participants’ answers to the medical conditions questionnaire, “Have you ever been informed that you have cancer or a malignant tumor?” “What type of cancer was it?” Only participants who identified colon and rectal cancer were noted.

### Temporal points for data collecting

Anthropometric data, including WC, height, and weight, were collected during participants’ visits to the Mobile Examination Center (MEC). These measurements were obtained at specific time points within the survey period. Information regarding colorectal cancer was collected through self-reported questionnaires. Notably, the time of cancer diagnosis was not directly correlated with the timing of the anthropometric data collection. Consequently, the study design operated under the assumption that the anthropometric data reflected the participants’ health status at the time of the survey. Any case of colorectal cancer diagnosed subsequent to the survey visit was regarded as a later-occurring event.

### Covariates

This study evaluated covariates such as age, gender, ethnicity, BMI, ratio of family income to poverty (PIR), education level (≤high school, >high school), smoking, drinking status, exercise, and diabetes. Smoking: Classified as yes (smoked ≥100 cigs in life) or no based on the questionnaire. Drinking status: Divided into drinkers (affirmative to “consume 4/5 cups or more/day”) and non-drinkers. Exercise: Those doing vigorous exercise etc. for ≥10 min weekly are exercisers. Diabetes: Determined by self-report of diagnosis or blood sample with glycated Hb ≥ 6.5%. Data was collected via NHANES’ standardized questionnaires & interview protocols. The waist-to-height ratio (WHtR) was computed by taking the waist circumference, measured in centimeters, and dividing it by the height, which was also measured in the same unit of centimeters. In terms of the body roundness index (BRI), the following formula was employed to compute it: BRI = 364.2−365.5 × (1 − [wc(m)/2π]^2^ / [0.5 × height(m)]^2^)^½^ (14, 15).

### Statistical analysis

Due to the multi-stage probability sampling in NHANES, sample weights were incorporated into the analytical procedure. At the outset, a univariate analysis was carried out to compare the individual baseline characteristics of the participants. The presentation of continuous variables was as the weighted mean ± standard deviation (SD). To evaluate the differences in average levels between groups, *t*-tests were used. Categorical variables, on the other hand, were expressed as actual frequencies and weighted percentages, and chi-square tests were utilized to compare their distributions among different groups. To analyze the connection between ABSI and CRC, logistic regression models were employed. The outcomes were presented as odds ratios (OR) and 95% confidence intervals (CI). Three separate models were devised: Model 1, which remained unadjusted for covariates; Model 2, which was adjusted for age, gender, and race; and Model 3, which was adjusted for all relevant elements. The same procedure was applied to look into the correlation among ABSI quartiles, considering the lowest quartile as the reference. We also used restricted cubic spline curves (RCS) to explore potential non-linear relationships. Furthermore, subgroup analyses were carried out with respect to diverse factors including age, gender, exercise, diabetes, and BMI. The aim of these subgroup analyses was to investigate the disparities and possible variations within different subgroups as well as to evaluate the interactions among them. Finally, after carrying out the adjustment for all covariates, an examination was conducted on the receiver operating characteristic (ROC) curves of the subjects. The area under the curve (AUC) of ABSI was compared with that of other factors such as the BRI, the WHtR, WC, BMI, and weight. Additionally, the AUC of ABSI was compared by categorizing the individuals based on age (≥60 and < 60). All of the statistical analyses were performed with the utilization of R (version 4.2.0) and EmpowerStats software (version 4.2). It was considered that a *p* value less than 0.05 signified a statistically significant difference.

## Results

### Baseline characterization

The baseline characteristics of the two groups of study participants divided according to whether they had colorectal cancer are shown in Table 1. The group without CRC had 40,628 participants, while the group with CRC included 370 participants. The mean age of the group with colorectal cancer was 67.40 ± 13.57 years. In the group free from colorectal cancer, the average ABSI was 0.081 ± 0.005, whereas in the group having colorectal cancer, it was 0.084 ± 0.004. Notable differences were observed between these two groups regarding a diverse range of factors, namely age, BMI, WC, WHtR, ABSI, BRI, ethnicity, education level, drinking habits, diabetes status, exercise frequency, and smoking status (all with *p* < 0.05). Table 2 is categorized based on the ABSI quartiles. The findings indicated a progressive rise in mean age from 36.90 ± 13.26 years in the Q1 group to 56.49 ± 15.51 years in the Q4 group. A substantial disparity existed among the four tertile groups regarding the presence or absence of CRC (*p* < 0.001). 0.13% (24) of the Q1 cohort had colorectal cancer; 0.49% (59) of the Q2 cohort had CRC; 1.07% (136) of the Q3 cohort had colorectal cancer; and 1.15% (151) of the Q4 cohort had CRC. With the rise in quartiles, there was a corresponding increase in the incidence of CRC cases. This may indicate that specific factors pertaining to the quartiles are maybe linked to the chance of acquiring CRC. Moreover, substantial disparities were seen among the ABSI quartile groups concerning other characteristics (all *p* < 0.001).

**Table 1:** Baseline characteristics of the study population classified according to colorectal cancer.

Variable	Participants without colorectal cancer (*N* = 40,628)	Participants with prior colorectal cancer (*N* = 370)	*p*-value
Age, years	45.32 ± 16.16	67.40 ± 13.57	<0.001
PIR	3.00 ± 1.64	2.86 ± 1.57	0.449
Weight, kg	82.95 ± 21.77	82.01 ± 18.88	0.392
Height, cm	168.74 ± 10.05	167.14 ± 9.78	0.176
BMI, kg/m^2^	29.05 ± 6.92	29.21 ± 5.94	0.037
WC, cm	98.83 ± 16.23	102.52 ± 13.95	<0.001
WHtR	0.59 ± 0.10	0.61 ± 0.08	<0.001
ABSI	0.081 ± 0.005	0.084 ± 0.004	<0.001
BRI	5.31 ± 2.27	5.90 ± 2.05	<0.001
Gender, *N* (weighted %)			0.692
Male	19,564 (48.71)	182 (45.63)	
Female	21,064 (51.29)	188 (54.37)	
Race, *N* (weighted %)			<0.001
Mexican American	6,183 (8.57)	20 (2.59)	
Other Hispanic	3,711 (5.98)	26 (3.27)	
Non-Hispanic White	17,352 (65.65)	225 (77.24)	
Non-Hispanic Black	8,811 (11.65)	75 (9.65)	
Other Race	4,571 (8.15)	24 (7.25)	
Levels of education, *N* (weighted %)			0.007
≤High school	18,676 (38.79)	196 (41.99)	
>High school	21,952 (61.21)	174 (58.01)	
Drinking status, *N* (weighted %)			<0.001
Yes	13,902 (30.26)	157 (35.39)	
No	26,726 (69.74)	213 (64.61)	
Diabetes, *N* (weighted %)			<0.001
Yes	6,029 (10.85)	115 (25.32)	
No	34,599 (89.15)	255 (74.68)	
Exercise, *N* (weighted %)			<0.001
Yes	10,917 (31.29)	34 (9.92)	
No	29,711 (68.71)	336 (90.08)	
Smoking, *N* (weighted %)			<0.001
Yes	17,667 (43.64)	206 (54.66)	
No	22,961 (56.36)	164 (45.34)	

**Table 2:** Baseline characteristics of study subjects classified by quartiles of ABSI score.

Variable	Q1 (*N* = 10,250)	Q2 (*N* = 10,249)	Q3 (*N* = 10,249)	Q4 (*N* = 10,250)	*p*-value
Age, years	36.90 ± 13.26	42.43 ± 14.14	48.16 ± 15.62	56.49 ± 15.51	<0.001
PIR	3.00 ± 1.65	3.11 ± 1.64	2.99 ± 1.65	2.87 ± 1.62	<0.001
Weight, kg	79.70 ± 20.98	83.09 ± 21.51	84.97 ± 22.99	84.39 ± 21.09	<0.001
Height, cm	167.59 ± 9.75	169.18 ± 9.94	169.00 ± 10.14	169.25 ± 10.35	<0.001
BMI, kg/m^2^	28.42 ± 7.41	28.94 ± 6.76	29.62 ± 7.12	29.30 ± 6.11	<0.001
WC, cm	90.18 ± 14.94	97.55 ± 15.17	101.93 ± 14.47	107.46 ± 15.27	<0.001
WHtR	0.54 ± 0.09	0.58 ± 0.09	0.60 ± 0.09	0.64 ± 0.09	<0.001
BRI	4.31 ± 2.13	5.09 ± 2.14	5.67 ± 2.09	6.42 ± 2.22	<0.001
Gender, *N* (weighted %)					<0.001
Male	4,138 (39.43)	5,016 (50.90)	5,144 (51.79)	5,448 (53.67)	
Female	6,112 (60.57)	5,233 (49.10)	5,105 (48.21)	4,802 (46.33)	
Race, *N* (weighted %)					<0.001
Mexican American	1,224 (7.63)	1707 (9.74)	1711 (9.45)	1,561 (7.10)	
Other Hispanic	929 (6.71)	983 (6.31)	983 (5.99)	842 (4.60)	
Non-Hispanic White	3,663 (59.18)	4,204 (64.72)	4,375 (66.65)	5,335 (73.82)	
Non-Hispanic Black	3,268 (18.13)	2,133 (10.63)	2015 (10.06)	1,470 (6.83)	
Other Race	1,166 (8.35)	1,222 (8.60)	1,165 (7.85)	1,042 (7.65)	
Levels of education, *N* (weighted %)					<0.001
≤High school	3,868 (32.42)	4,451 (36.37)	5,046 (42.09)	5,507 (45.82)	
>High school	6,382 (67.58)	5,798 (63.63)	5,203 (57.91)	4,743 (54.18)	
Drinking status, *N* (weighted %)					<0.001
Yes	2,916 (25.16)	3,152 (26.91)	4,053 (36.15)	3,938 (34.08)	
No	7,334 (74.84)	7,097 (73.09)	6,196 (63.85)	6,312 (65.92)	
Diabetes, *N* (weighted %)					<0.001
Yes	591 (4.12)	1,074 (7.34)	1791 (13.32)	2,688 (20.91)	
No	9,659 (95.88)	9,175 (92.66)	8,458 (86.68)	7,562 (79.09)	
Exercise, *N* (weighted %)					<0.001
Yes	4,325 (46.10)	3,072 (33.95)	2,140 (24.79)	1,414 (16.79)	
No	5,925 (53.90)	7,177 (66.05)	8,109 (75.21)	8,836 (83.21)	
Smoking, *N* (weighted %)					<0.001
Yes	3,568 (35.27)	4,177 (40.78)	4,698 (46.80)	5,430 (54.01)	
No	6,682 (64.73)	6,072 (59.22)	5,551 (53.20)	4,820 (45.99)	
Colorectal cancer, *N* (weighted %)					<0.001
No	10,226 (99.87)	10,190 (99.51)	10,113 (98.93)	10,099 (98.85)	
Yes	24 (0.13)	59 (0.49)	136 (1.07)	151 (1.15)	

### The relationship between ABSI and CRC

**Figure 2:**
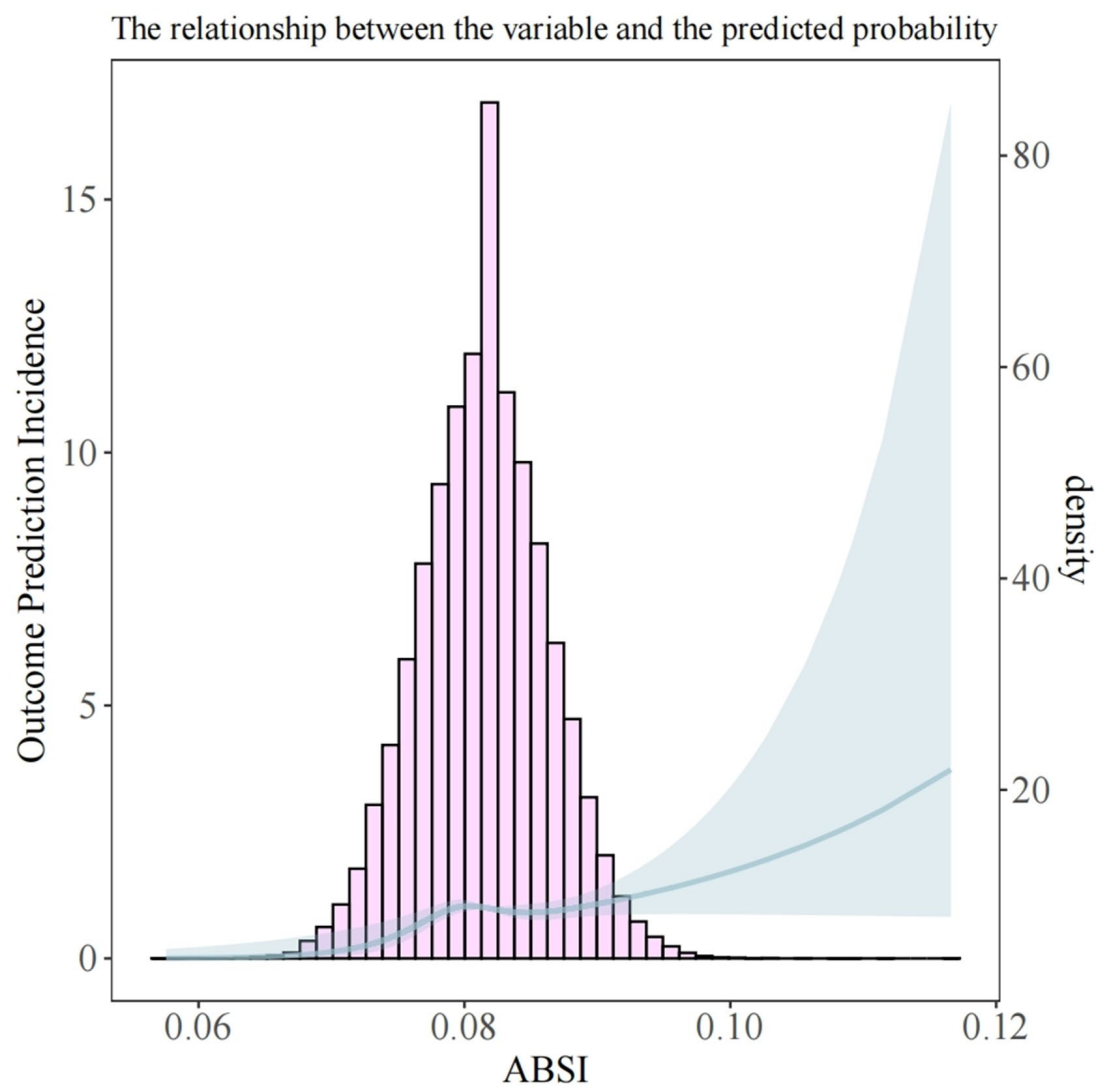
RCS analysis of the association between ABSI and CRC.

To evaluate the association between ABSI and CRC, we employed logistic regression analysis (Table 3). When ABSI was analyzed as a continuous variable without any adjustments, the OR for the association between ABSI and CRC was found to be 1.12 (95% CI: 1.10–1.14, and *p* < 0.001). In Model 2, after making adjustments for gender, age, and race, OR = 1.05 [95% CI: 1.03–1.07], and *p* < 0.001. After fully adjusting for covariables, OR = 1.03 [95% CI: 1.01–1.05], with *p* = 0.018. Each of the above-cited models showed a positive connection between ABSI and CRC. Furthermore, we converted ABSI into a four-category variable for the purpose of sensitivity analysis, which provided further support for this finding. In Model 3, compared to the Q1 group, the Q2 group had (OR = 1.60 [95% CI: 0.99–2.59], *p* = 0.054). The Q3 group had (OR = 2.50 [95% CI: 1.60–3.91], *p* < 0.001). And the Q4 group had (OR = 1.88 [95% CI: 1.19–2.96], *p* = 0.006). Trend analyses showed that all 3 models had statistical significance. In Model 1, the trend *p* value was <0.001. In Model 2, the trend *p* value was also <0.001. In Model 3, the trend *p* value was = 0.017. This further substantiates that when the ABSI quartiles rise, there may be a progressively increasing trend in the incidence of CRC. Furthermore, RCS analysis confirmed a significant non-linear correlation between ABSI and CRC. Figure 2 shows a significant overall trend (*P* for overall = 0.014) and a non-linear association (*P* for nonlinear = 0.030) between ABSI and CRC. When the ABSI value was approximately 0.082, the curve shifted from a relatively gentle upward trend to a steeper one. This indicates that as the ABSI value increases, the risk of CRC also rises.

**Table 3:** Association between ABSI and CRC.

Exposure	Model 1 OR (95%CI)	*p*-value	Model 2 OR (95%CI)	*p*-value	Model 3 OR (95%CI)	*p* value
ABSI	1.12 (1.10, 1.14)	<0.001	1.05 (1.03, 1.07)	<0.001	1.03 (1.01, 1.05)	0.018
ABSI quartiles						
Q1 (0.058, 0.078)	Ref		Ref		Ref	
Q2 (0.078, 0.082)	2.47 (1.53, 3.97)	<0.001	1.71 (1.06, 2.77)	0.028	1.60 (0.99, 2.59)	0.054
Q3 (0.082, 0.085)	5.73 (3.71, 8.85)	<0.001	2.96 (1.90, 4.61)	<0.001	2.50 (1.60, 3.91)	<0.001
Q4 (0.085, 0.117)	6.37 (4.14, 9.81)	<0.001	2.42 (1.55, 3.78)	<0.001	1.88 (1.19, 2.96)	0.006
*P* for trend	<0.001		<0.001		0.017	

Model 1: No covariates were adjusted.

Model 2: Adjust for age, gender, race.

Model 3: Adjust age, gender, race, PIR, BMI, levels of education, smoking, drinking status, exercise, diabetes.

### Subgroup analysis

**Figure 3:**
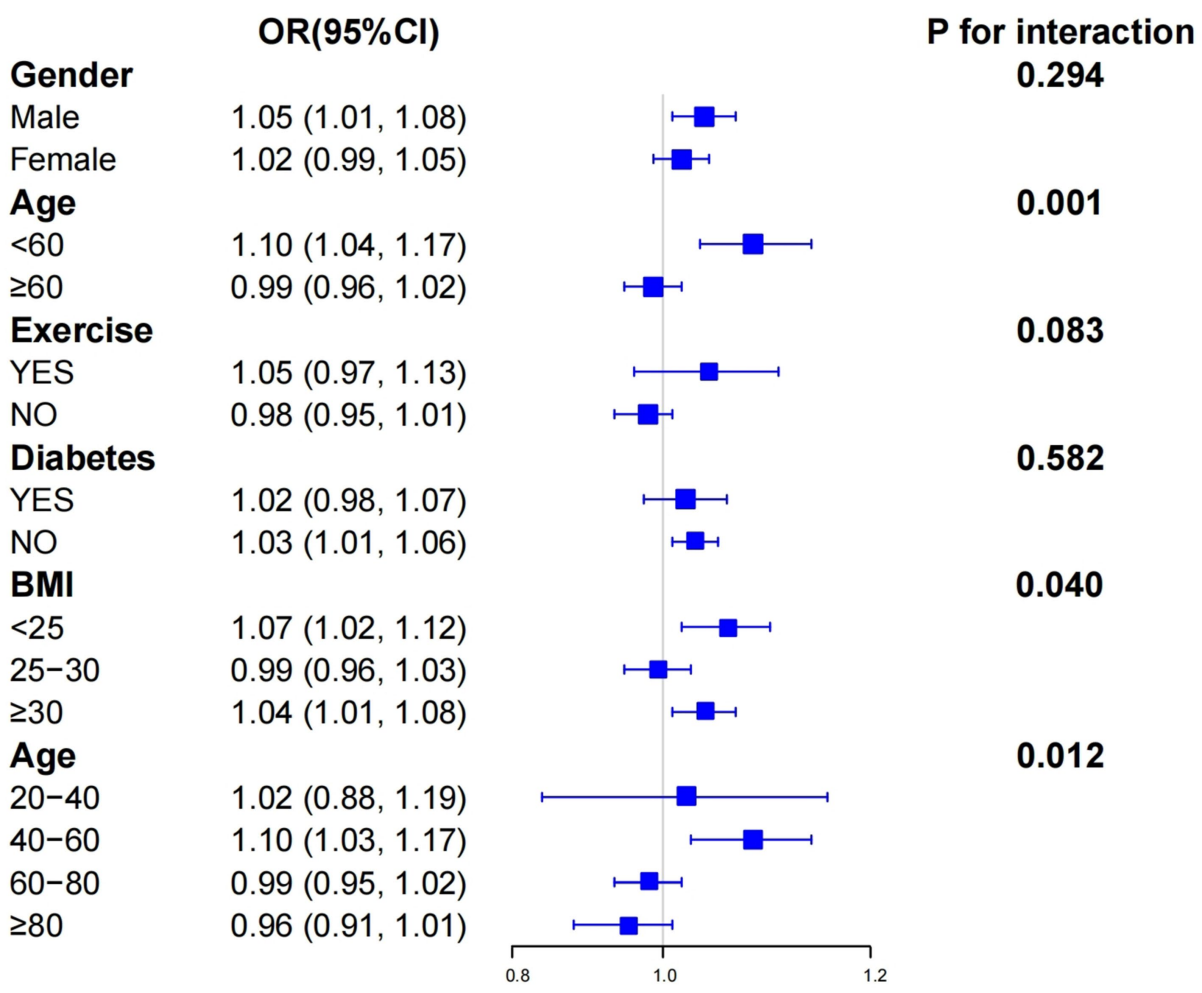
Subgroup analysis between ABSI and CRC.

In the subgroup analysis, we delved deeper into exploring how gender, age, exercise, diabetes, and BMI impact the relationship between ABSI and CRC, as depicted in Figure 3. A significant interaction was detected between different age subgroups and the correlation between ABSI and CRC. Specifically, for those with an age less than 60, the OR was 1.10 [with a 95% CI of 1.04–1.17]; while for those aged 60 or above, the OR was 0.99 [95% CI: 0.96–1.02], and the interaction *p* value was 0.001. This clearly shows that age has a substantial influence on the relationship between ABSI and CRC. To further explore the role of age, we conducted additional analyses using four age groups: 20–40 years old, 40–60 years old, 60–80 years old, and 80 years old and above. The findings indicated that the correlation between ABSI and CRC was most pronounced in the 40 to 60-year-old age demographic, exhibiting an OR of 1.10 [95% CI: 1.03–1.17] and an interaction *p* value of 0.012. Moreover, it is noteworthy that BMI also affects the relationship between ABSI and CRC, presenting a certain level of complexity. For individuals with a BMI less than 25, the OR was 1.07 [95% CI: 1.02–1.12]; and for those with a BMI of 30 or higher, the OR was 1.04 [95% CI: 1.01–1.08], with an interaction *p* value of 0.040. In contrast, no notable interactions were detected in the remaining categories since all of the interaction *p* values within these instances were higher than 0.05.

### ROC curve analysis of ABSI vs. other anthropometric indicators

**Figure 4:**
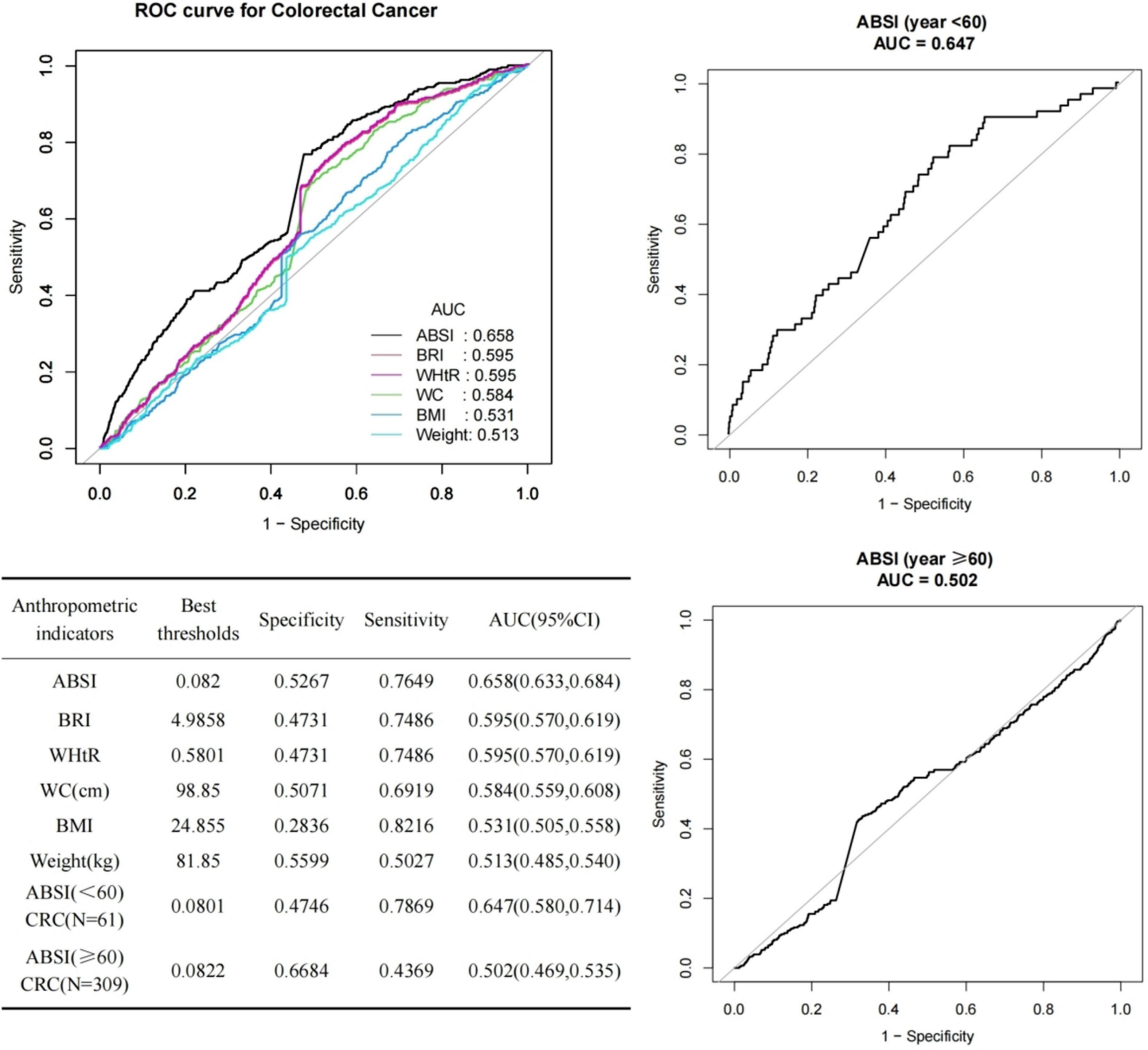
ROC curves for ABSI prediction of CRC with multiple anthropometric indices and age stratification.

We compared the diagnostic efficacy of the ABSI with that of other anthropometric measures regarding the prevalence of CRC. The findings are shown in Figure 4. In the case of ABSI, the optimal cut-off value was determined to be 0.082, accompanied by a specificity of 0.5267 and a sensitivity of 0.7649. Additionally, it can be seen from Figure 4 that the performance of ABSI outperformed that of BRI, WHtR, WC, BMI, and Weight (AUC = 0.658). We further classified the subjects based on their age. For those who were younger than 60 years old, the value of 0.0801 was established as the optimal cut-off value of the ABSI. At this cut-off, the specificity was 0.4746 and the sensitivity was 0.7869. In the case of individuals who were 60 years old or older, the value of 0.0822 was established as the optimal cut-off value of the ABSI. Here, the specificity was 0.6684 and the sensitivity was 0.4369. The AUC of ABSI for those younger than 60 years old (AUC = 0.647) was significantly higher than that for those aged 60 years or older (AUC = 0.502), as depicted in Figure 4. The performance of various anthropometric indicators in predicting colorectal cancer differs. On the whole, ABSI holds certain value in predicting colorectal cancer, particularly demonstrating a relatively high sensitivity among individuals younger than 60 years old. Meanwhile, other indicators such as BRI, WHtR, WC, BMI, and Weight also possess some predictive capabilities for colorectal cancer to varying degrees (with AUC > 0.5).

## Discussion

This study represents the first investigation to comprehensively analyze the relationship between ABSI and CRC in a broad population across the United States, leveraging the NHANES dataset. We additionally included and examined the most recent NHANES data from 2003 to 2023. The results indicated that ABSI is positively correlated with an increased incidence of CRC. This correlation remained significant even after taking all variables into account. In the subgroup analyses carried out according to age and BMI, a significant interaction between the ABSI and CRC was detected. In ROC analysis, we discovered that the association of ABSI with CRC exceeded that of other obesity indices, indicating that ABSI may be more precise than other obesity-related metrics in predicting CRC. The data revealed that the predictive capacity and accuracy of the ABSI for CRC were higher in individuals under 60 years of age than in those who were 60 years old or older. These findings suggest directions for further research and propose that, in clinical practice, ABSI should be considered as an auxiliary tool for risk assessment, especially for specific population subgroups such as those aged less than 60 years, and those with varying BMI categories. This would enable the formulation of more targeted preventive interventions.

Among the various factors associated with cancer development, visceral obesity is more likely to be a key carcinogenic factor than overall fat (16). Research has indicated that visceral obesity is a prevalent disease that is typically accompanied by low adiponectin levels. Moreover, it is a firmly recognized risk factor for CRC (17). This is because visceral obesity triggers a series of complex pathophysiological changes that further increase the risk of cancer (18–20). For instance, several pro-inflammatory cytokines like tumor necrosis factor-alpha (TNF-*α*) and interleukin-6 (IL-6) are secreted by visceral adipose tissue (5). These cytokines keep the immune system activated continuously, thereby leading to a state of chronic inflammation. Prolonged chronic inflammation can result in recurrent damage and repair of the colorectal mucosal epithelial cells, heightening the probability of genetic alterations and hence facilitating the onset and progression of colorectal cancer (21, 22). Furthermore, adipose tissue, especially the visceral fat within it, can cause the levels of insulin and insulin-like growth factor-I (IGF-I) in the serum to go up. It can also prompt the release of leptin and adiponectin. These alterations significantly contribute to the etiology of cancer (23). Computed tomography (CT) and magnetic resonance imaging (MRI) are commonly used to measure the distribution of visceral fat. Nevertheless, these techniques are expensive, take a lot of time, and are not appropriate for large-scale epidemiologic studies (24). Consequently, a straightforward clinical anthropometric index, the ABSI, is suggested for assessing visceral fat. The ABSI is founded on the notion of anisotropic growth in calculating BMI and has successfully developed BMI-independent alternatives to waist indices (10). From a statistical perspective, the ABSI has no dependence on height, BMI, and WC, and more accurately represents visceral adiposity than conventional anthropometric measures. Anthropometric measurements more accurately represent visceral obesity compared to conventional anthropometric assessments (25). In contrast to other novel anthropometric measures like the BRI, the ABSI has something to do with increased cancer risk in numerous spots (26), whereas the BRI has infrequently been connected with cancer (15). This suggests that the ABSI is more centered on measuring visceral fat, which is highly relevant to the incidence of cancer.

In recent years, the ABSI has gained increasing recognition for measuring visceral fat accumulation and its impact (27, 28). Studies have also demonstrated a positive relation between ABSI and the risk of neurological diseases (13), cardiovascular diseases (7), and all-cause mortality (29). Moreover, an increasing amount of research is exploring the connection between the ABSI and cancer risk. Studies regarding the UK Biobank cohort have shown that ABSI is markedly linked to a greater risk of five particular malignancies, among which are lung cancer and CRC, and the overall risk of developing cancer as well (26). A cohort research from the Guangzhou Biobank in China, with a 14-year follow-up, manifested a positive linkage between ABSI and an augmented risk of CRC (30). An analysis that combined and involved collaboration of 11 Australian cohorts showed that ABSI correlates with total cancer and CRC in both genders, but not with prostate cancer (31). Nevertheless, research on the American NHANES database finds that higher ABSI levels are closely linked to prostate cancer risk and its related death rates (25, 32). This indicates that ABSI may be subject to certain limitations when directly applied to populations with different ethical backgrounds, and the current evidence regarding the relationship of ABSI to CRC remains comparatively limited. Consequently, we undertook a comprehensive examination of the relationship between ABSI and CRC within the American populace. Our study results show that ABSI has a positive relation with a higher risk of CRC, particularly in specific demographics where this association is more significant. Furthermore, to explore the relationship between ABSI and CRC more comprehensively, we conducted an in-depth analysis and comparison of relevant studies. A study based on NHANES data from 1999 to 2018 investigated the association between ABSI and the risk of CRC among patients with metabolic syndrome. It was found that, in this patient group, a higher ABSI was significantly associated with an increased risk of developing CRC (33). In contrast, our study, based on a broader population in the United States, has further expanded the scope of research on the relationship between ABSI and CRC. Although the research subjects differ, both studies highlight the importance of ABSI in assessing the risk of CRC. Moreover, the results of our study, to some extent, corroborate those of the previous study, indicating that ABSI may be an important predictor of CRC, not restricted by the single factor of metabolic syndrome. Christakoudi et al. revealed the association between body shape phenotypes and imaging-based body composition measurements, as well as their relationship with colon cancer (34). Their research demonstrated that body shape phenotypes defined by ABSI and hip index (HI) were closely related to body composition and could reflect differences therein, such as variations in the distribution of visceral adipose tissue (VAT) and abdominal subcutaneous adipose tissue (ASAT). These differences were associated with the risk of colon cancer. Our study also detected an association between ABSI and CRC, further supporting the crucial role of body composition, especially abdominal fat distribution, in the pathogenesis of CRC. Rontogianni et al. provided new evidence for the causal relationship between body shape indices and the risk of CRC through a Mendelian randomization study (35). They discovered a potential positive causal correlation between the allometric index ABSI, waist-hip index (WHI), and CRC, which was independent of traditional waist-circumference indices and BMI. The results of our study echo theirs, further strengthening the reliability of ABSI as a risk predictor for CRC. Simultaneously, this also provides a direction for more in-depth research on causal mechanisms in the future.

In this study, we also found that the association between ABSI and CRC risk was more pronounced, especially in the subgroup of individuals under 60 years old (40–60 years old). The subgroup analysis accurately pinpointed the age range of 40–60 years old, where the association was significant, revealing the close connection between ABSI and CRC risk in this age group. In addition, the ROC analysis, as a supplementary tool for exploring the overall age-related trends in this study, showed that ABSI had a certain value in predicting CRC risk, especially in the population under 60 years old, where it exhibited high sensitivity. The results of these two analyses are closely related and refer to each other. From a more macroscopic perspective, that is, the grouping of ages ≥60 years old and < 60 years old, the ROC analysis helps us to grasp the overall trend of the predictive ability of ABSI with age, further confirming the findings of the subgroup analysis and emphasizing the importance of age as a modifying factor when considering the relationship between ABSI and CRC risk. This finding is consistent with what Krakauer et al. pointed out regarding the high correlation between ABSI and age, and is in line with previous research results, indicating that within this age range, ABSI might be an effective risk predictor (33). Specifically, the higher metabolic rate in younger people may amplify the impact of abdominal fat distribution on subsequent health outcomes (36). Additionally, unhealthy lifestyle behaviors, such as sedentary habits and poor dietary choices, are more prevalent among middle-aged individuals (37). This may further exacerbate the association between ABSI and the risk of CRC. As people age, the clinical manifestations of elderly patients tend to be more complex, often accompanied by multiple comorbidities. In this age group, the influence of ABSI still exists, but it may be masked by other factors, such as genetic factors and past medical history. Elderly patients may experience vague symptoms such as weight loss and decreased appetite, which are often attributed to aging rather than cancer (30). This thus influences the correlation between ABSI and CRC. These combined factors can explain the differences in the strength of the association between ABSI and CRC observed in different age subgroups.

To date, no study has explored the correlation between ABSI and the risk of CRC in the general population of the United States. The large sample size is another advantage of this study. However, we cannot overlook the limitations of the research. This study was conducted based on the cross-sectional survey data of NHANES. This approach may introduce temporal bias in the reference time points between anthropometric measurements and CRC. The relationship between anthropometric data and the risk of CRC may change over time, and the cross-sectional design restricts the ability to clarify causal relationships. Thus, it is impossible to rule out the possibility of reverse causation or bidirectional causation. Secondly, even after adjusting for some potential confounding variables in the statistical analysis, there may still be undetected confounding factors, which could affect the accuracy of the results. Finally, NHANES did not collect imaging and pathological data from its participants. Therefore, relying on self-reported CRC data may introduce information bias. Especially among the elderly population, due to memory decline, insufficient disease awareness, or limited access to medical care, they may not accurately report their CRC status, leading to an underestimation of the number of CRC cases and thus affecting the accuracy of the research results. Future research should adopt more objective cancer diagnosis methods, such as integrating clinical examination data and medical records, to reduce the bias caused by self-reporting. Meanwhile, more detailed survey plans can be designed for different age groups to improve the accuracy of the data.

## Conclusion

This study finds that a bigger ABSI is tied to a higher CRC risk, especially for people under 60 years of age (40–60 years) and those with different BMI types. Compared to traditional anthropometric measurements, ABSI has a unique value in predicting CRC. Notably, it shows enhanced sensitivity in those aged less than 60 years. Future studies should the precise mechanisms of ABSI and CRC to formulate novel strategies for decreasing incidence rates. Furthermore, public health education must be enhanced to promote food regulation, consistent physical activity to diminish visceral fat buildup, and the endorsement of healthy lifestyles to mitigate the risk of CRC.

## Data Availability

Publicly available datasets were analyzed in this study. This data can be found here: http://www.cdc.gov/nchs/nhanes.htm.

## Glossary

ABSI: A body shape index
CRC: Colorectal cancer
NHANES: National Health and Nutrition Examination Survey
BMI: Body mass index
WC: Waist circumference
OR: Odds ratio
CI: Confidence interval
ROC: Receiver operating characteristic
PIR: Ratio of family income to poverty
WHtR: Waist-to-height ratio
BRI: Body roundness index
TNF-*α*: Tumor necrosis factor-alpha
IL-6: Interleukin-6
GF-I: Insulin-like growth factor-I
CT: Computed tomography
MRI: Magnetic resonance imaging
RCS: Restricted cubic spline
ASAT: Abdominal subcutaneous adipose tissue
VAT: Visceral adipose tissue
HI: Hip index
WHI: Waist-hip index
